# Inhibition of Cutaneous TRPV3 Channels by Natural Caffeic Acid for the Alleviation of Skin Inflammation

**DOI:** 10.3390/molecules29163728

**Published:** 2024-08-06

**Authors:** Guoji Zhang, Liqin Wang, Yaxuan Qu, Shilun Mo, Xiaoying Sun, Kewei Wang

**Affiliations:** 1 Department of Pharmacology, School of Pharmacy, Qingdao University Medical College, 1 Ningde Road, Qingdao 266073, China; 2 Department of Natural Medicinal Chemistry and Pharmacognosy, School of Pharmacy, Qingdao University Medical College, 1 Ningde Road, Qingdao 266073, China; 3 Institute of Innovative Drugs, Qingdao University, 38 Dengzhou Road, Qingdao 266021, China

**Keywords:** caffeoyl analogues, caffeic acid, skin inflammation, TRPV3

## Abstract

Natural caffeic acid (CA) and its analogues have been studied for their potential applications in the treatment of various inflammatory and infectious skin diseases. However, the molecular mechanism underlying the effects of the CA remains largely unknown. Here, we report that CA and its two analogues, caffeic acid phenethyl ester (CAPE) and caffeic acid methyl caffeate (CAMC), inhibit TRPV3 currents in their concentration- and structure-dependent manners with IC_50_ values ranging from 102 to 410 μM. At the single-channel level, CA reduces the channel open probability and open frequency without alteration of unitary conductance. CA selectively inhibits TRPV3 relative to other subtypes of thermo-TRPs, such as TRPA1, TRPV1, TRPV4, and TRPM8. Molecular docking combined with site-specific mutagenesis reveals that a residue T636 in the Pore-loop is critical for CA binding to TRPV3. Further *in vivo* evaluation shows that CA significantly reverses TRPV3-mediated skin inflammation induced by skin sensitizer carvacrol. Altogether, our findings demonstrate that CA exerts its anti-inflammatory effects by selectively inhibiting TRPV3 through binding to the pocket formed by the Pore-loop and the S6. CA may serve as a lead for further modification and identification of specific TRPV3 channel inhibitors.

## 1. Introduction

The warmth-sensitive and Ca^2+^-permeable transient receptor potential vanilloid 3 (TRPV3) is robustly expressed in the skin, playing a critical role in skin sensation, skin barrier formation, hair growth, and vasodilation [1,2,3]. The gain-of-function mutations in the TRPV3 channel not only lead to rodent hairlessness and dermatitis but also cause human-inherited skin disease Olmsted syndrome, characterized by keratoderma, dermatitis, hair loss, and severe itch [4,5,6]. In the lesional epidermis of atopic dermatitis patients and pruritic burn scars, the expression of TRPV3 is markedly upregulated [7,8,9]. Suppression of TRPV3 channel function by gene knockout and pharmacological inhibitors has been shown to alleviate dermatitis and pruritus [10,11,12]. These investigations highlight the potential therapeutic value of pharmacological inhibition of TRPV3 for the therapy of skin diseases.

Currently, the identification of TRPV3 channel inhibitors mainly comes from repurposing drugs and medicinal plants, such as a local anaesthetic dyclonine [13] and an antispasmodic agent, flopropione [14]. These not only provide useful pharmacological tool molecules for channel research but also suggest the potential for repurposing as anti-dermatitis and anti-pruritus agents. In addition, many natural TRPV3 inhibitors have been reported, such as monanchomycin B [15], urupocidin A [15], osthole [16,17], forsythoside B [18], verbascoside [19], citrusinine-II [20], scutellarein [21], isochlorogenic acid A and isochlorogenic acid B [22], α-mangostin [23], honokiol and magnolol [24]. Therefore, it is rational to identify selective inhibitors of TRPV3 from natural products.

**Figure 1:**
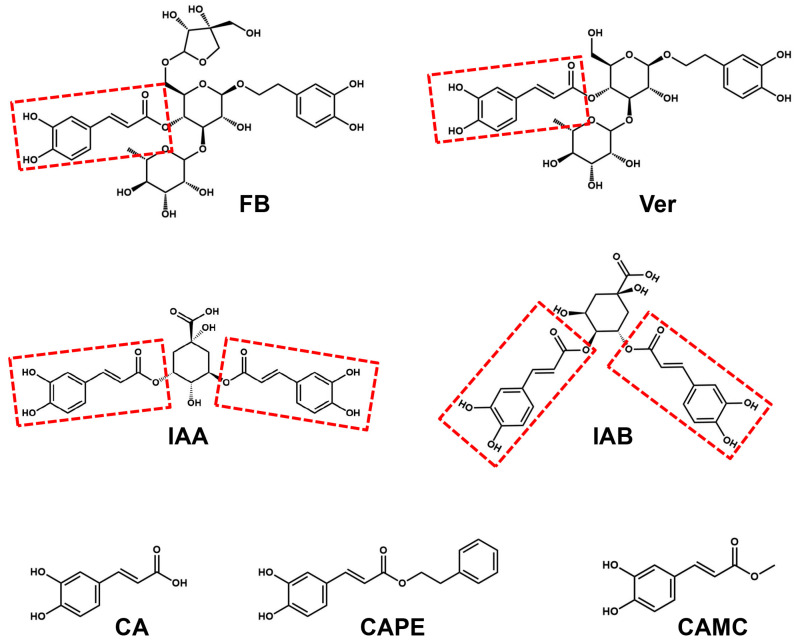
The chemical structures of forsythiaside B (FB), verbascoside (Ver), isochlorogenic acid A (IAA), isochlorogenic acid B (IAB), caffeic acid (CA), caffeic acid phenethyl ester (CAPE), and caffeic acid methyl caffeate (CAMC).

Natural caffeic acid (CA) and its analogues, such as caffeic acid phenethyl ester (CAPE) and caffeic acid methyl caffeate (CAMC), are phenolic compounds found in fruits, propolis, coffee, and many medicinal plants, and they exhibit a wide range of biological activities, including anti-inflammatory, anti-pruritic, anti-microbial effects and protection against ultraviolet irradiation [25,26,27,28]. However, the molecular mechanism underlying the effects of these caffeoyl analogues remains largely unknown. It is also of interest that many natural TRPV3 inhibitors, including forsythiaside B, verbascoside, isochlorogenic acid A, and isochlorogenic acid B, contain caffeioyl groups (Figure 1). This suggests that caffeoyl analogues likely act on TRPV3 to exert their biological activities. To test this hypothesis, we selected three natural caffeoyl analogues that mainly differed in the substituent of acrylic acid at the fourth position of the phenyl ring and tested their effects on the inhibition of TRPV3.

In this study, we found three caffeoyl analogues that inhibit TRPV3 currents in concentration- and structure-dependent manners. CA specifically inhibits the TRPV3 channel over other thermo-TRPs, such as TRPA1, TRPV1, TRPV4, and TRPM8. CA binds to the pocket formed by the Pore-loop and the S6, and it also effectively alleviates skin inflammation. These findings indicate that CA could be developed as a lead or an agent to improve TRPV3-related skin diseases.

## 2. Results

### 2.1. Concentration- and Structure-Dependent Inhibition of hTRPV3 Currents by Caffeoyl Analogues

**Figure 2:**
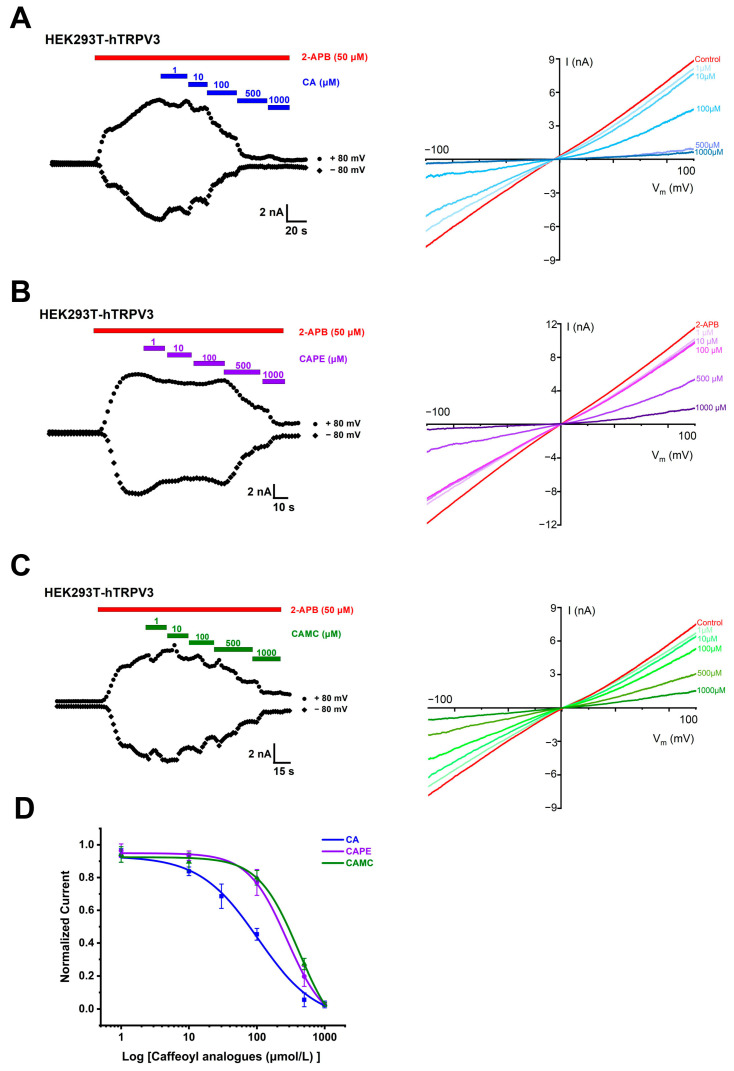
Concentration- and structure-dependent inhibition of TRPV3 currents by caffeoyl analogues. (**A**–**C**) Left panel, inhibition of whole-cell currents of human TRPV3 (hTRPV3) channel activated by agonist 2-aminoethyl diphenylborinate (2-APB, 50 μM, red bar) and increasing concentrations of caffeic acid (CA, blue bar), caffeic acid phenethyl ester (CAPE, purple bar), or caffeic acid methyl caffeate (CAMC, green bar) from 1 to 1000 μM. Right panel, current–voltage curves of hTRPV3 in response to voltage ramps from −100 to +100 mV from the left panel after the addition of 50 μM 2-APB and co-addition of CA, CAPE, or CAMC from 1 to 1000 μM. (**D**) The concentration-dependent inhibition of hTRPV3 by caffeoyl analogues at +80 mV was analysed by Hill equation fitting, with IC_50_ value of 102.1 ± 19.7 μM (CA, *n* = 5), 276.7 ± 41.9 μM (CAPE, *n* = 5), and 409.8 ± 57.9 μM (CAMC, *n* = 5). Data are expressed as the mean ± SD.

To assess the effects of caffeoyl analogues on TRPV3 channels, we carried out whole-cell current recordings of TRPV3 currents expressed in human embryonic kidney (HEK) 293T cells. We started testing the overexpression of TRPV3 in HEK293T cells 24 h after transient transfection of human TRPV3 cDNA. Consistent with the previous literature [29], TRPV3 protein is abundantly expressed in HEK293T cells transiently transfected with hTRPV3 (Figure S1). We proceeded to test the effect of caffeic acid (CA) and its two analogues, caffeic acid phenethyl ester (CAPE) and caffeic acid methyl caffeate (CAMC), on hTRPV3. The perfusion of CA at concentrations ranging from 1 to 1000 μM resulted in the concentration-dependent inhibition of hTRPV3 currents activated by agonist 50 μM 2-APB with IC_50_ values of 102.1 ± 19.7 μM (Figure 2A,D). CAPE and CAMC inhibited whole-cell TRPV3 currents induced by 2-APB (50 μM) in a concentration-dependent manner with the right-shifted IC_50_ values of 276.7 ± 41.9 μM and 409.8 ± 57.9 μM, respectively (Figure 2B–D). These results demonstrate the concentration- and structure-dependent inhibition of hTRPV3 currents by CA and its analogues CAPE and CAMC.

### 2.2. Inhibition of Single TRPV3 Channel by Caffeic Acid

**Figure 3:**
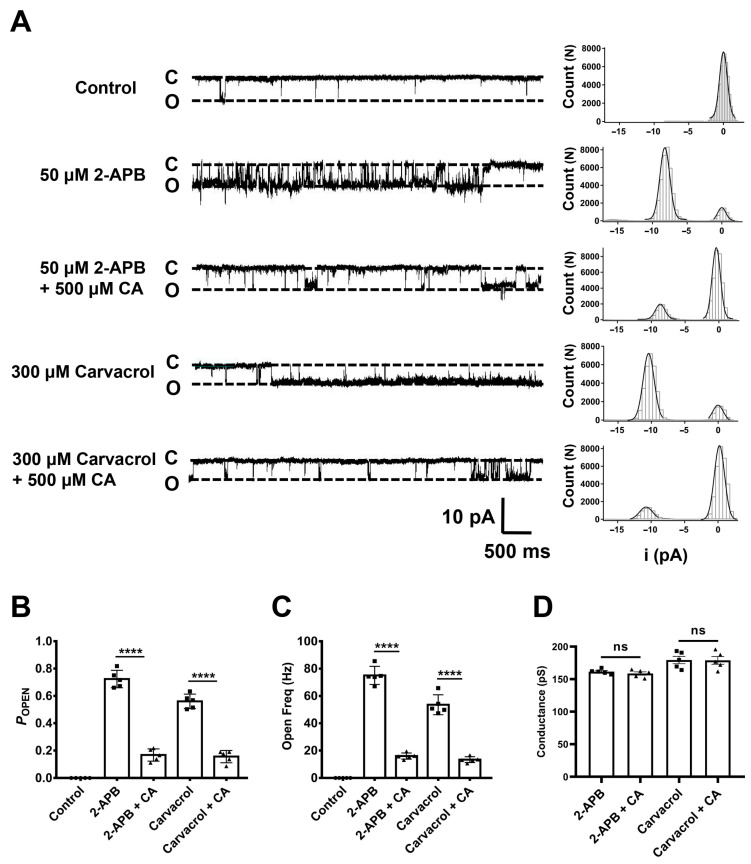
Reduction of hTRPV3 single-channel open probability by caffeic acid. (**A**) Left panel, representative single-channel current traces recorded at −60 mV in inside-out configurations before and after the addition of different TRPV3 agonists (50 μM 2-APB or 300 μM carvacrol) and co-application with 500 μM caffeic acid (CA). All-point amplitude histograms of single-channel currents in 5 s were shown in their right panels. Dotted lines indicate the closed channel state (C) and the opened channel state (O), respectively. (**B**) Summary of the average open probability (*P*_OPEN_) values of hTRPV3 single channel in the presence of control (circle), different TRPV3 agonists (square) and co-application with CA (triangles) (*n* = 5, **** *p* < 0.0001, by unpaired *t* test). (**C**) Summary of hTRPV3 single-channel open frequency (Freq) after exposure to control (circle), different TRPV3 agonists (square) and co-application with CA (triangles) (*n* = 5, **** *p* < 0.0001, by unpaired *t* test). (**D**) Summary of hTRPV3 single channel conductance after exposure to different TRPV3 agonists (square) and co-application with CA (triangles) (*n* = 5, ns, no significance, by unpaired *t* test). Data are expressed as the mean ± SD.

To further confirm the inhibitory effect of CA on the TRPV3 channel, we recorded the single channels in an inside-out configuration. The activation of TRPV3 channels by agonist 2-APB (50 μM) shows an increase in single-channel open probability (*P*_OPEN_) to 0.72 ± 0.06 and open frequency (Freq) to 75.05 ± 6.63 Hz with single-channel conductance of 161.6 ± 3.8 pS, whereas CA at 500 μM reduced *P*_OPEN_ to 0.16 ± 0.04 and open Freq to 15.96 ± 2.25 Hz without any significant alteration of channel conductance of 158.7 ± 5.8 pS (Figure 3A–D). Similarly, agonist carvacrol at 300 μM also increased the single-channel *P*_OPEN_ to 0.56 ± 0.05 and open Freq to 53.55 ± 7.27 Hz with a single-channel conductance of 179.4 ± 12.8 pS, whereas CA at 500 μM reduced *P*_OPEN_ to 0.15 ± 0.04 and open Freq to 13.31 ± 2.13 Hz without any significant alteration of channel conductance of 178.8 ± 13.2 pS (Figure 3A–D). These results confirm that CA acts directly on individual TRPV3 channels by reducing the channel open probability and open frequency without altering its unitary conductance.

**Figure 4:**
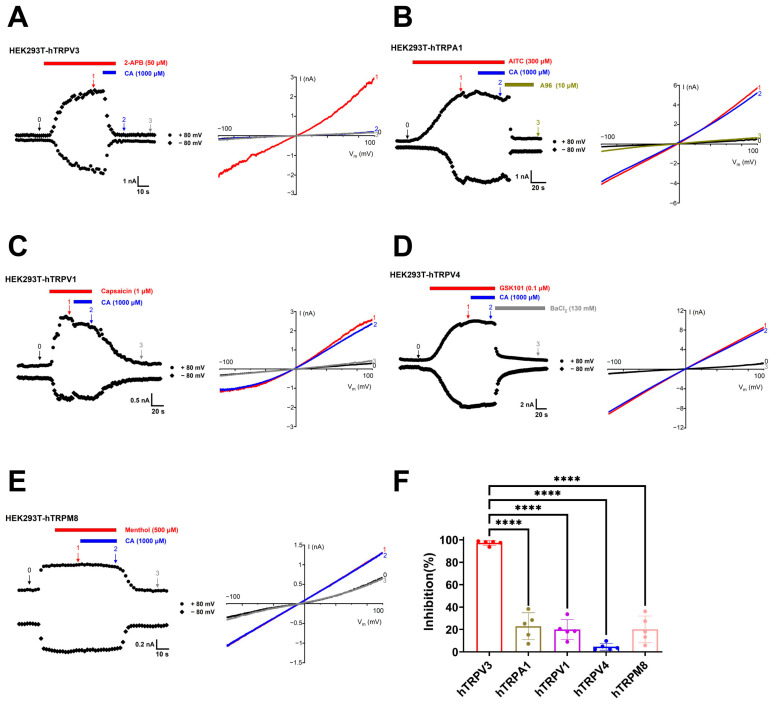
Selectivity of caffeic acid for TRPV3 over TRPA1, TRPV1, TRPV4, and TRPM8 channels. (**A**) Left panel, whole-cell current recordings of human TRPV3 (hTRPV3) channels expressed in HEK293T cells in response to 50 μM 2-APB (red bar) and co-application of 1000 μM caffeic acid (CA, blue bar). Right panel, current–voltage curves of hTRPV3 in response to voltage ramps from −100 to +100 mV under control condition (0) after addition of 50 μM 2-APB (1) and co-addition of 1000 μM CA (2) and washout (3). (**B**) Left panel, whole-cell current recordings of human TRPA1 (hTRPA1) channels expressed in HEK293T cells in response to 300 μM TRPA1 agonist allyl isothiocyanate (AITC, red bar) and co-application of 1000 μM CA (blue bar) and inhibited by TRPA1 antagonist A96 (10 μM, brown bar). Right panel, current–voltage curves of hTRPA1 in response to voltage ramps from −100 to +100 mV under control condition (0) after addition of 300 μM AITC (1) and co-addition of 1000 μM CA (2) and inhibited by 10 μM A96 (3). (**C**) Left panel, whole-cell current recordings of human TRPV1 (hTRPV1) channels expressed in HEK293T cells in response to 1 μM TRPV1 agonist capsaicin (red bar) or co-application of 1000 μM CA (blue bar) and washout. Right panel, current–voltage curves of hTRPV1 in response to voltage ramps from −100 to +100 mV under control condition (0) after addition of 1 μM capsaicin (1) and co-addition of 1000 μM CA (2) and washout (3). (**D**) Left panel, whole-cell current recordings of human TRPV4 (hTRPV4) channels expressed in HEK293T cells in response to 0.1 μM TRPV4 agonist GSK1016790A (GSK101, red bar) and co-application of 1000 μM CA (blue bar) and inhibited by 130 mM BaCl_2_ (grey bar). Right panel, current–voltage curves of hTRPV4 in response to voltage ramps from −100 to +100 mV under control condition (0) after addition of 0.1 μM GSK101 (1) and co-addition of 1000 μM CA (2) and inhibited by 130 mM BaCl_2_ (3). (**E**) Left panel, whole-cell current recordings of human TRPM8 (hTRPM8) channels expressed in HEK293T cells in response to 500 μM TRPM8 agonist menthol (red bar) or co-application of 1000 μM CA (blue bar) and washout. Right panel, current–voltage curves of hTRPM8 in response to voltage ramps from −100 to +100 mV under control condition (0) after addition of 500 μM menthol (1) and co-addition of 1000 μM CA (2) and washout (3). (**F**) Summary of average current inhibition of hTRPV3 (red), hTRPA1 (brown), hTRPV1 (purple), hTRPV4 (blue), and hTRPM8 (pink) channels by 1000 μM CA, (*n* = 5, **** *p* < 0.0001, by unpaired *t* test). Data are expressed as the mean ± SD.

To determine the selectivity of CA, we tested its effects on other thermo-TRP channels, such as TRPA1, TRPV1, TRPV4, and TRPM8 channels expressed in HEK293T cells. CA at 1000 μM inhibited the human TRPV3 current induced by 2-APB at 50 μM about 97.2 ± 2.0% (Figure 4A,F). CA at 1000 μM only inhibited the human TRPA1 current induced by allyl isothiocyanate (AITC) at 300 μM about 22.9 ± 12.0% (Figure 4B,F) and inhibited the current of human TRPV1 caused by 1 μM capsaicin about 20.0 ± 8.8% (Figure 4C,F). Similarly, 1000 μM CA caused a slight inhibition of the human TRPV4 current caused by 0.1 μM GSK1016790A about 4.6 ± 3.1% (Figure 4D,F) and inhibited human TRPM8 current caused by 500 μM menthol about 20.1 ± 11.9% (Figure 4E,F). These results indicate that CA is a relatively selective inhibitor of the TRPV3 channel over other subtypes, such as TRPA1, TRPV1, TRPV4, and TRPM8 channels.

### 2.3. Identification of TRPV3 Residues Crucial for Caffeic Acid Binding

**Figure 5:**
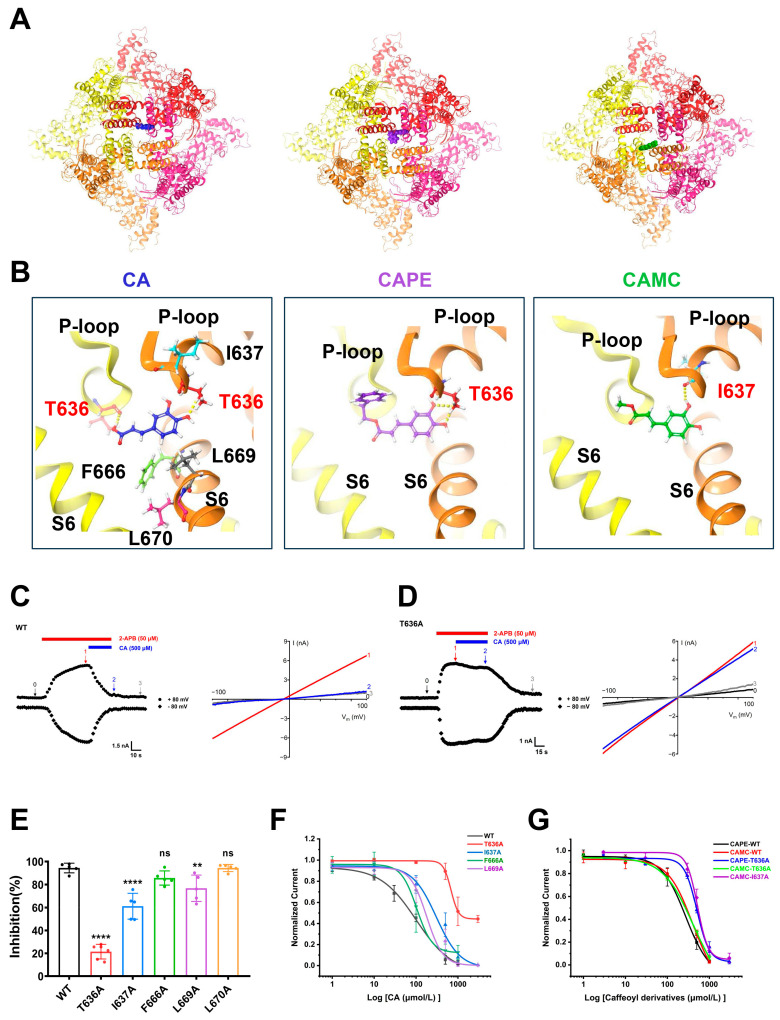
Binding site and key residues of caffeic acid and its analogues binding to TRPV3 channels. (**A**) The top-down view of a putative pocket for caffeic acid (CA, blue), caffeic acid phenethyl ester (CAPE, purple), and caffeic acid methyl caffeate (CAMC, green) binding to mouse TRPV3 structure (PDB ID code: 6DVY) from docking. The four subunits of the tetramer are distinguished in four different colours. (**B**) The side view of CA (blue), CAPE (purple), and CAMC (green) in the pocket formed by Pore-loop (*p*-loop) and the S6 segment with the key residues T636 or I637 through the hydrogen bonds (yellow dotted line). (**C**,**D**) Left panel, representative whole-cell recordings of wild-type (WT) hTRPV3 (**C**) and T636A (**D**) mutant expressed in HEK293T cells in the presence of 50 μM 2-APB alone (red bar) and co-application of 500 μM caffeic acid (CA, blue bar). Right panel, current–voltage curves of WT hTRPV3 and T636A mutant in response to voltage ramps from −100 to +100 mV under control condition (0) after addition of 50 μM 2-APB (1) and co-addition of 500 μM CA (2) and washout (3). (**E**) Summary for WT hTRPV3 (black) or mutants (T636A, red; I637A, blue; F666A, green; L669A, purple; L670A, orange) channel currents inhibition by 500 μM CA (*n* = 5; ns, no significance; ** *p* < 0.01; **** *p* < 0.0001; by unpaired *t* test). (**F**) The concentration-dependent inhibition of WT hTRPV3 and mutations outward currents by CA at +80 mV was analysed by Hill equation fitting, with IC_50_ value of 102.1 ± 19.7 μM (WT from Figure 2D, *n* = 5), 104.5 ± 4.8 μM (F666A, *n* = 5), 179.7 ± 59.9 μM (L669A, *n* = 5), 305.6 ± 61.1 μM (I637A, *n* = 5), >1000 μM (T636A, *n* = 4). (**G**) The concentration-dependent inhibition of WT hTRPV3 and mutations outward currents by CAPE and CAMC at +80 mV was analysed by Hill equation fitting, with IC_50_ value of 276.7 ± 41.9 μM (CAPE-WT from Figure 2D, *n* = 5), 409.8 ± 57.9 μM (CAMC-WT from Figure 2D, *n* = 5), 506.3 ± 57.3 μM (CAPE-T636A, *n* = 5), 416.7 ± 45.1 μM (CAMC-T636A, *n* = 4), and 522.0 ± 43.8 μM (CAMC-I637A, *n* = 5). Data are expressed as the mean ± SD.

To further validate the binding pocket between CA and TRPV3, we utilized Schrödinger’s Glide model for molecular docking of CA and its analogues CAPE and CAMC into the cryo-EM structure of mTRPV3 (PDB: 6DVY). The docking predicted that CA, CAPE, and CAMC are confined in the central cavity pocket formed by the Pore-loop and the S6 segment (Figure 5A), consistent with our previous finding for caffeoyl analogues isochlorogenic acid A and isochlorogenic acid B binding to TRPV3 [22]. CA is mainly recognized by the residues T636, I637, F666, L669, and L670, with a docking score of −5.4. Within this pocket, two hydroxyl groups of the phenyl ring and acrylic acid of CA bind to the residue T636 from two *p*-loops through two hydrogen bonds. CAPE is recognized by the residue T636 through two hydrogen bonds with a docking score of −5.1, while CAMC is recognized by the residue I637 through one hydrogen bond with a docking score of −4.8 (Figure 5B).

To further validate the key residues critical for CA binding to TRPV3, we performed site-directed mutagenesis on the residues, including T636, I637, F666, L669, and L670. Whole-cell recordings showed that mutating T636, I637, and L669 to alanine significantly diminished TRPV3 inhibition by CA compared to WT channel currents. Conversely, the mutations F666A and L670A showed no significant difference in sensitivity to CA-mediated TRPV3 inhibition compared to the WT (Figure 5C–E). We also determined the IC_50_ values of CA for T636A, I637A, F666A, and L669A mutant channels. Among the determined IC_50_ values, CA exhibited the lowest potency with an IC_50_ of 1000 μM for the T636A mutant, approximately 10-fold less potent than the WT (Figure 5F). Mutating T636 into alanine led to reduced potencies of CAPE with IC_50_ values of 506.3 ± 57.3 μM. Conversely, the T636A mutant showed no significant alteration in sensitivity to CAMC-mediated TRPV3 inhibition with IC_50_ of 416.7 ± 45.1 μM, as compared to the IC_50_ of 409.8 ± 57.9 μM in WT. In contrast, mutating I637 into alanine (I637A) led to reduced potencies of CAMC with an IC_50_ value of 522.0 ± 43.8 μM (Figure 5G). These results demonstrate the critical role of the T636 residue in the pocket for CA binding to the TRPV3 channel.

### 2.4. Caffeic Acid Alleviates Skin Inflammation Induced by Skin Sensitizer Carvacrol

**Figure 6:**
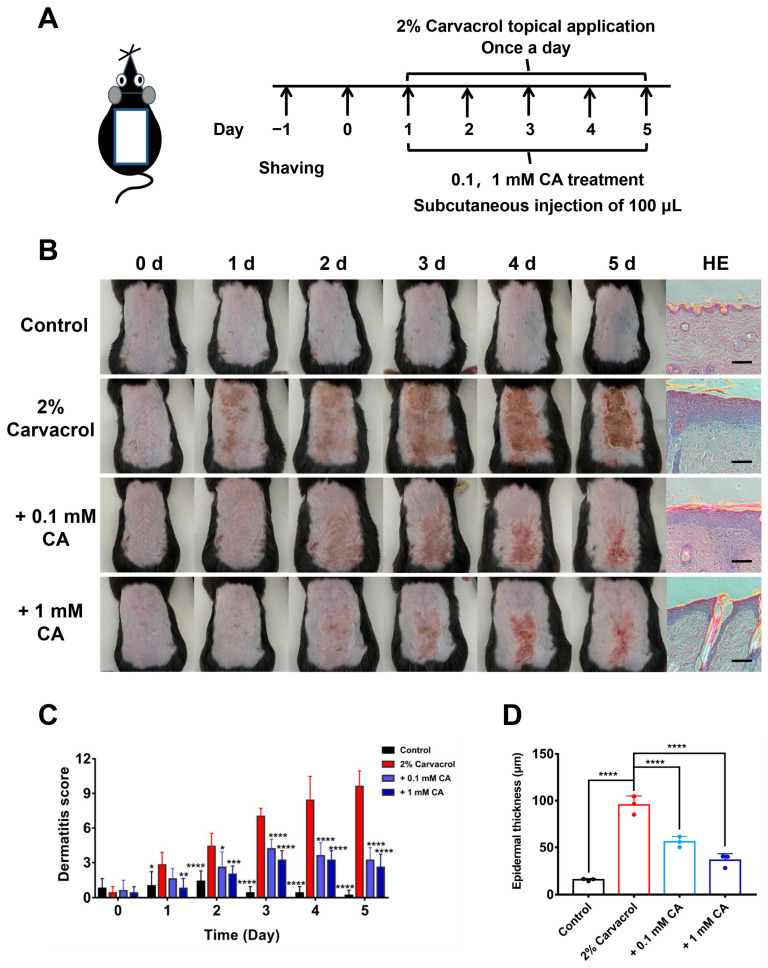
Attenuation of dermatitis induced by skin sensitizer carvacrol by caffeic acid. (**A**) Schematic drawing of experimental procedures for generation of the mouse dermatitis model of dorsal skin by topical applications of skin sensitizer carvacrol (2%) and subcutaneous injections of caffeic acid (CA) at different concentrations (0.1, 1 mM) for 5 consecutive days. (**B**) Phenotypic features and histologic images of H&E staining of dorsal skin tissue sections before and after topical applications of carvacrol (2%) for 5 consecutive days with and without subcutaneous injections of different concentrations CA. Scale bar = 100 μm for histologic images. (**C**) Dermatitis scores of mice in different groups treated with or without CA at different concentrations for 5 consecutive days from B (*n* = 5, * *p* < 0.05, ** *p* < 0.01, *** *p* < 0.001, **** *p* < 0.0001, by two-way ANOVA, followed by the Bonferroni’s test). (**D**) Summary of epidermal thickness of mouse dorsal skin sections (*n* = 3, **** *p* < 0.0001, by one-way ANOVA, followed by the Dunnet’s test). Data are expressed as the mean ± SD.

Overactive TRPV3 function by skin sensitizer carvacrol causes TRPV3-mediated cutaneous inflammation [11,21]. We, therefore, generated a mouse model of dorsal skin inflammation induced by topical applications of skin sensitizer carvacrol at 2% concentration for 5 consecutive days (Figure 6A). Topical carvacrol resulted in a time-dependent development of dorsal skin inflammation as compared with the vehicle control (vehicle: 30% ethanol and 70% saline). In contrast, subcutaneous injections of CA at different concentrations (0.1 and 1 mM) alleviated the skin inflammation and significantly reduced the dermatitis scores, as compared with the carvacrol model group (Figure 6B,C). We further carried out histological examinations of dorsal skin tissue sections and found that subcutaneous injections of CA at different concentrations (0.1 and 1 mM) reduced epidermal thickness induced by carvacrol, as compared with the carvacrol model group (Figure 6B,D), which are consistent with above phenotypic observations. These results indicate that CA alleviates TRPV3-mediated skin inflammation induced by the skin sensitizer carvacrol.

## 3. Materials and Methods

### 3.1. Animals

Adult male C57BL/6J mice (aged 6–8 weeks, weighing 20 ± 3 g) purchased from Vital River Laboratory Animal Technology Co., Ltd. (Beijing, China). All experimental mice were acclimated for at least one week before experiments for their adaptation to new experimental environment where the temperature was maintained at 22 ± 2 °C with 12 h light and dark circulation per day. Mice had free access to water and food. All *in vivo* experimental protocols are approved by the Institutional Animal Care and Use Committee of Qingdao University Health Science Center (protocol code QDU-AEC-2023092), approval date 10 March 2023 (Qingdao, China).

### 3.2. Compounds

The natural compounds caffeic acid (CA, MW: 180.15), caffeic acid methyl caffeate (CAMC, MW: 194.18), and caffeic acid phenethyl ester (CAPE, MW: 284.31) were purchased from Tauto Biotech Co., Ltd. (Shanghai, China). Compounds 2-aminoethoxydiphenylborate (2-APB), carvacrol, capsaicin, menthol, barium chloride (BaCl_2_), GSK1016790A (GSK101), and allyl isothiocyanate (AITC) were purchased from Sigma Aldrich (St. Louis, MO, USA). The purity of each standard compound was no less than 98% by high-performance liquid chromatography analysis. All compounds are prepared in DSMO stock solutions. For patch-clamp recording, compounds were diluted with extracellular fluid used for perfusion. For skin inflammation models, carvacrol was added to 2% using a saline solution containing 30% ethanol, and the other compounds were diluted using saline.

### 3.3. Cell Cultures

HEK293T cell line was obtained from the Cell Resource Center, Peking Union Medical College (Beijing, China). HEK293T cells were cultured in Dulbecco’s modified Eagle medium (DMEM, Gibco, ThermoFisher, Grand Island, NY, USA) supplemented with 10% foetal bovine serum (FBS, PAN-Biotech, Aidenbach, Bayern, Germany) at 37 °C and 5% CO_2_. HEK293T cells were digested with trypsin and inoculated onto glass slides for 24 h before transfection with 2.5 µg of hTRPV3 cDNAs (NM_145068.4), hTRPA1 (NM_007332.3), hTRPV1 (NM_080704.3), hTRPV4 (NM_021625.5), and hTRPM8 (NM_024080.5) using Lipofectamine 2000 (Invitrogen, Carlsbad, CA, USA). Recordings of HEK293T cells were performed 18 h after transfection.

### 3.4. Electrophysiological Recordings

Patch clamp recordings were conducted using an EPC10 amplifier powered and analysed by Patchmaster software (HEKA Harvard, Holliston, Church Hill, TN, USA). The borosilicate glass pipettes were fabricated using a vertical micropipette puller (PC-100, Narishige, Tokyo, Japan) to achieve the tip resistances of 3–5 MΩ for whole-cell recording and 5–10 MΩ for single-channel recording when filled with pipette solution. Pipette solution and bath solution both contained (in mM) 130 NaCl, 3 HEPES, and 0.2 EDTA (pH = 7.4) [14]. Whole-cell patch clamp recording showed that the membrane potential was maintained at 0 mV; currents were recorded at a slope voltage ramp from −100 to +100 mV for 500 ms and analysed at ±80 mV. For inside-out patch recordings of single channels, membrane potential was held at 0 mV before a voltage of −60 mV to monitor the opening of single channels. Single channel currents were sampled at 10 kHz and filtered at 2.0 kHz. All recordings were conducted at room temperature of 22 ± 2 °C, and data were analysed using Igor Pro (Wave-metrics, Lake Oswego, OR, USA) and Origin 8.6 (OriginLab, Northampton, MA, USA).

### 3.5. Molecular Docking and Site-Directed Mutagenesis

Molecular docking was performed using Schrödinger Glide (Maestro software suite 2015, Schrödinger, New York, NY, USA). Small molecules CA, CAPE, and CAMC were drawn using ChemBioDraw Ultra 14.0 (Cambridge Soft, Cambridge, MA, USA) and optimized for docking using the built-in program Ligprep in Maestro. The TRPV3 EM-structure (PBD code: 6DVY) was obtained from the Protein Data Bank and docked using the standard docking module SP according to Maestro’s standard procedure. The binding pocket between ligand and TRPV3 was selected using Glide based on the reported inhibitor binding sites for TRPV3 and ranked based on the obtained scores. All TRPV3 site-directed mutations were generated using the Mut Express II Fast Mutagenesis Kit V2 according to the manufacturer’s instructions. All mutants were confirmed by sequencing for correct generation of mutation.

### 3.6. Mouse Models of Dermatitis Induced by Skin Sensitizer Carvacrol

Mice were placed in a gas anaesthesia device (SurgiVet, Smiths Medical, Minneapolis, MN, USA) for anaesthesia before the hair on the back was removed with a razor, followed by the application of an appropriate amount of hair removal cream to gently shave off the remaining hair. Carvacrol (2%) was topically applied to mouse dorsal skin once a day for 5 consecutive days. Different concentrations of CA (0.1, 1 mM) in 100 μL were prepared in saline solution before subcutaneous injection into the mouse dorsal skin 30 min before topical application of 2% carvacrol once a day for 5 days.

### 3.7. Evaluation of Skin Lesions

The severity of skin lesions is evaluated based on four symptoms: (1) erythema/bleeding, (2) scar/dryness, (3) oedema, (4) scratch/erosion. The score for each symptom ranges from 0 to 3 (none, 0; mild, 1; moderate, 2; severe, 3). Dermatitis score is defined as the sum of individual scores, ranging from 0 to 12 points [11].

### 3.8. Histological Sections of Skin Tissues

Mouse dorsal skin tissues were dissected using scissors and forceps immediately after sacrifice and fixed with 4% paraformaldehyde overnight at 4 °C before being dehydrated in ethanol and embedded in paraffin. Paraffin-embedded tissues were sectioned and stained with haematoxylins and eosin (H&E). Stained sections were observed using bright-field microscopy (ECLIPSE Ti-S, Nikon, Tokyo, Japan) and CCD-camera (DS-Ri2, Nikon, Tokyo, Japan).

### 3.9. Statistical Analysis

All data are expressed as the mean ± standard deviation (SD). Unpaired *t*-test and one-way and two-way ANOVA followed by multiple-comparison test were used to evaluate statistical significance using GraphPad Prism 7.0 software (La Jolla, CA, USA). A value of *p* < 0.05 is considered statistically significant.

## 4. Discussion

By analysing the chemical structures of known TRPV3 inhibitors, we find that many natural inhibitors contain caffeioyl groups. In this study, we selected caffeic acid (CA) and its two analogues, caffeic acid phenethyl ester (CAPE) and caffeic acid methyl caffeate (CAMC), and tested their inhibitory effects on TRPV3 currents. These three caffeoyl chemicals are confined in the same binding pocket, and they share a common caffeic acid backbone but differ in their structural modifications. The molecular docking predicts that hydroxyl groups on CA and its two analogues are the key groups forming hydrogen bonds with TRPV3. CA with a free carboxylic acid group and two hydroxyl groups on the phenyal ring exhibits about three- or four-fold better potency than CAPE esterified with the hydroxyl group of phenethyl alcohol and CAMC esterified with a methyl group. CA (IC_50_ = 102.1 ± 19.7 μM) appeared to inhibit TRPV3 in low potency as compared to caffeoyl analogues, such as forsythiaside B (IC_50_ = 6.7 ± 0.7 μM) [18], verbascoside (IC_50_ = 14.1 ± 3.3 μM) [19], isochlorogenic acid A (IC_50_ = 2.7 ± 1.3 μM), and isochlorogenic acid B (IC_50_ = 0.9 ± 0.3 μM) [22], which could be related to the low number of hydroxyl groups. This structure–activity relationship may serve as a hint for further improvement by chemical modifications of CA for its derivatives.

In this study, our findings show that the F666A mutation showed no significant difference in sensitivity to CA-mediated TRPV3 inhibition compared to the WT type (Figure 5E,F). This mechanism of action of CA is distinct from TRPV3 inhibitors such as dyclonine, scutellarein, isochlorogenic acid A, and isochlorogenic acid B that primarily act on the channel residue F666 located at the S6-helix and critical for TRPV3 channel gating [13,21,22]. This may explain the worse inhibitory potency of CA with IC_50_ of approximately 100 μM and may serve as a strategy for enhanced potency of CA derivatives by increasing hydrogen bond interactions between the residue F666 of TRPV3 and CA derivatives.

Natural CA is a plant-derived compound with a wide range of pharmacological activities such as anti-oxidative, anti-inflammatory, anti-cancer, and anti-microbial effects [25,30,31,32,33]. CA has also been reported to inhibit histamine-induced intracellular calcium increase in the H1R/TRPV1 pathway and chloroquine-induced responses in the MRGPRA3/TRPA1 pathway, as well as to reduce scratching behaviour in mice induced by multiple itchy compounds. However, the effects of CA directly targeted the TRP channel have not been evaluated using patch clamp recordings [27]. In this study, we identified CA that selectively inhibits TRPV3 relative to other subtypes of thermo-TRPs, such as TRPV1 and TRPA1, using whole-cell and single-channel recordings. TRPV3 participates in signalling pathways associated with acute and chronic itch [34,35,36], making it more likely to be a molecular target of CA. In addition, numerous studies have reported that CA alleviates ultraviolet (UV)-induced skin injury by regulating multiple signalling pathways [37,38,39,40]. Recently, our laboratory has also reported the significant role of TRPV3 in UVB-induced skin damage [41]. We speculate that CA may prevent UV-induced inflammation and photocarcinogenesis by inhibiting TRPV3. However, further validation is needed to support this hypothesis.

In this study, the high concentration of CA and its two analogues was used *in vitro* and *in vivo* experiments. To make sure all the results are not influenced by toxicity, we evaluated the cellular toxicity of three caffeoyl chemicals in HEK293T cells. Consistent with the previous literature [27], the high concentration of CA (1 mM) treatment on HEK293T cells did not cause any noticeable cytotoxicity (Figure S2).

In conclusion, we demonstrate that three caffeoyl analogues inhibit TRPV3 channels in concentration- and structure-dependent manners. CA containing only one caffeioyl group inhibits the TRPV3 channel and alleviates skin inflammation. CA as the TRPV3 inhibitor not only explains the mechanistic insights into the anti-pruritic and anti-inflammatory effects of CA but also provides a lead for further optimization and identification of specific TRPV3 channel inhibitors.

## Data Availability

Data are contained within the article and Supplementary Materials.

## Supplementary Materials

The following supporting information can be downloaded at: https://www.mdpi.com/article/10.3390/molecules29163728/s1, Figure S1: The expressions of TRPV3 proteins in HEK293T cells transiently transfected with human TRPV3 cDNA; Figure S2: Effects of caffeoyl analogues on cell viability of HEK293T cells.

## Abbreviations

2-APB: 2-aminoethoxydiphenyl borate; AITC, allyl isothiocyanate; CA, caffeic acid; CAPE, caffeic acid phenethyl ester; CAMC, caffeic acid methyl caffeate; cryo-EM, cryoelectron microscopy; DMEM, Dulbecco’s modified Eagle’s medium; DMSO, dimethyl sulfoxide; FBS, foetal bovine serum; GSK101, GSK1016790A; H&E, haematoxylin and eosin; HEK293T, human embryonic kidney 293T; PDB, protein data bank; SD, standard deviation; TRP, transient receptor potential; TRPA, TRP ankyrin; TRPM, TRP melastatin; TRPV, TRP vanilloid; UV, ultraviolet; WT, white type.
