# Supplementary material for: Inhibition of Cutaneous TRPV3 Channels by Natural Caffeic Acid for the Alleviation of Skin Inflammation
Source: Molecules. 2024 Aug 6;29(16):3728. doi: 10.3390/molecules29163728 (PMC11357638; doi:10.3390/molecules29163728)
Supplement: Supplementary file 1 [file molecules-29-03728-s001.zip › molecules-3109014-supplementary.pdf]

molecules

MDPI

Supplementary information

Inhibition of Cutaneous TRPV3 Channels by Natural Caffeic Acid for the Alleviation of Skin Inflammation

Guoji Zhang [1], Liqin Wang [2], Yaxuan Qu [1], Shilun Mo [2], Xiaoying Sun [2,3,*] and KeWei Wang [1,3,*]

[1] Department of Pharmacology, School of Pharmacy, Qingdao University Medical College, 1 Ningde Road, Qingdao 266073, China
[2] Department of Natural Medicinal Chemistry and Pharmacognosy, School of Pharmacy, Qingdao University Medical College, 1 Ningde Road, Qingdao 266073, China
[3] Institute of Innovative Drugs, Qingdao University, 38 Dengzhou Road, Qingdao 266021, China
* Correspondence: xiaoyingsun@qdu.edu.cn (X.S.); wangkw@qdu.edu.cn (K.W.)

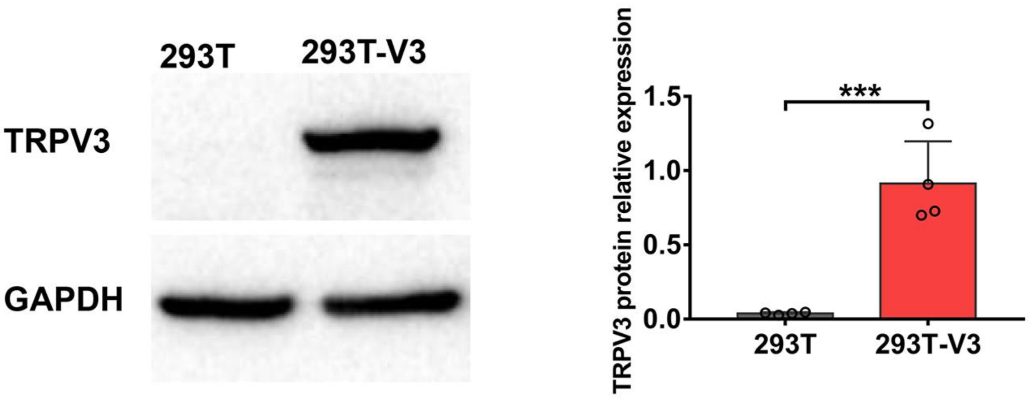

Figure S1. The expressions of TRPV3 proteins in HEK293T cells transiently transfected with human TRPV3 cDNA. Western blot analysis of TRPV3 protein expression level in HEK293T cells 24 hours after transient transfection of human TRPV3 cDNAs (n = 4, *** $P < 0.001$, by unpaired t test). Data are expressed as the mean ± SD.

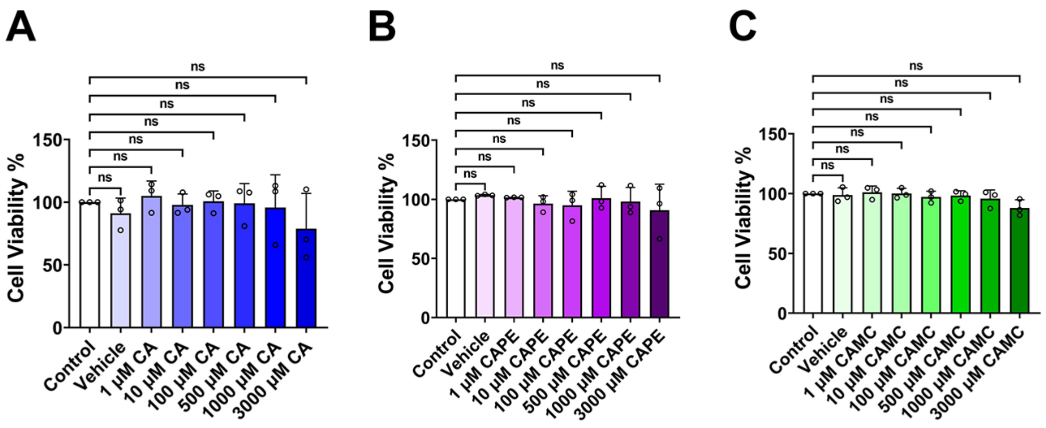

Figure S2. Effects of caffeoyl analogues on cell viability of HEK293T cells. (**A**–**C**) The cell viability of HEK293T cells was assessed using CCK-8 assay after 6 hours treatment with different concentrations of caffeic acid (CA), caffeic acid phenethyl ester (CAPE) and caffeic acid methyl caffeate

(CAMC). (n = 3, ns, no significance, by one-way ANOVA, followed by Dunnett's test). Data are expressed as the mean ± SD.
